# Supplementary material for: Generating a Small Shuttle Vector for Effective Genetic Engineering of Methanosarcina mazei Allowed First Insights in Plasmid Replication Mechanism in the Methanoarchaeon
Source: Int J Mol Sci. 2022 Oct 7;23(19):11910. doi: 10.3390/ijms231911910 (PMC9569500; doi:10.3390/ijms231911910)
Supplement: Supplementary file 1 [file ijms-23-11910-s001.zip › ijms-1933715-supplementary.pdf]

International Journal of
Molecular Sciences

MDPI

Supplementary Material

Generating a Small Shuttle Vector for Effective Genetic Engineering of *Methanosarcina mazei* Allowed First Insights in Plasmid Replication Mechanism in the Methanoarchaeon

Johanna Thomsen [1] and Ruth A. Schmitz [2,*]

This file includes:

Supplementary Figures S1 to S3

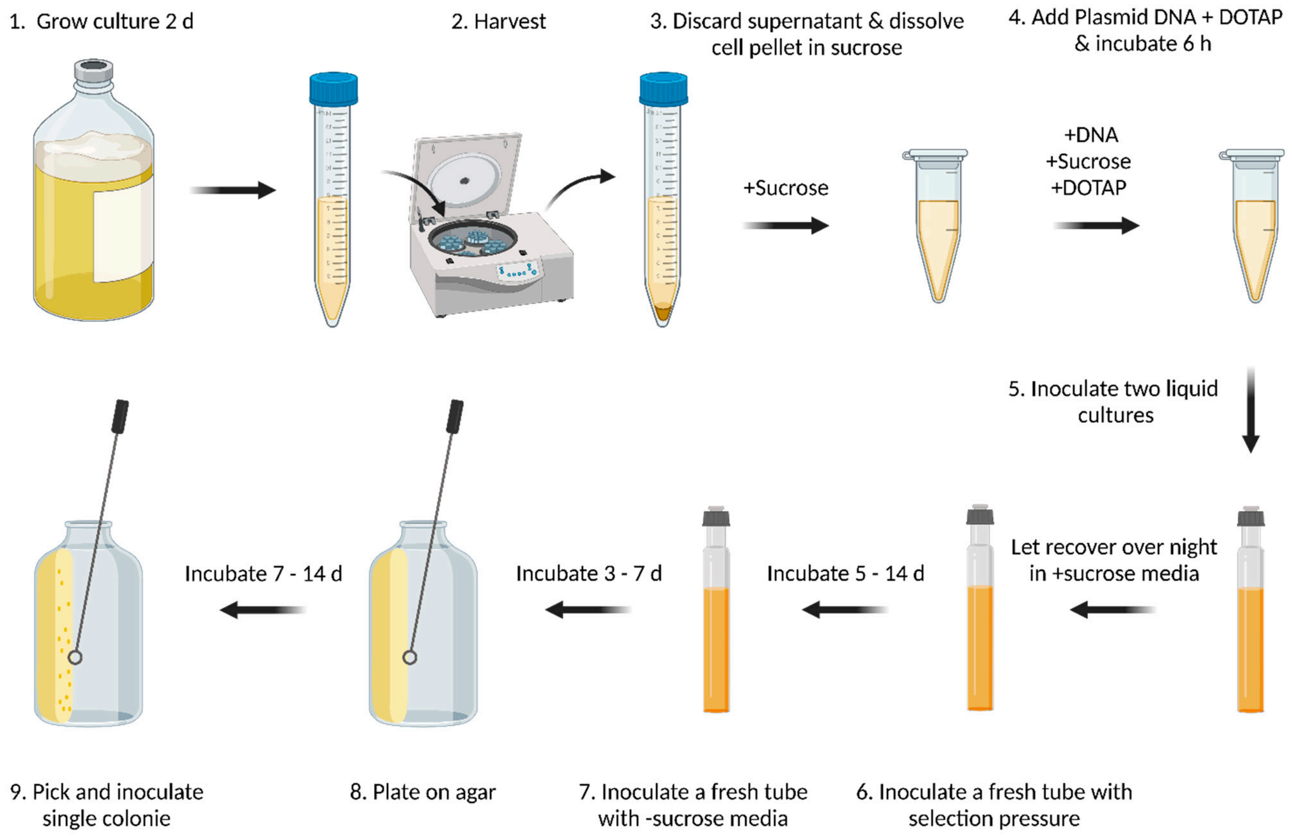

Figure S1. Liposome-mediated transformation and mutant selection in *Methanosarcina mazei*. Exponentially growing *M. mazei** cells (1) are harvested by centrifugation (2). The cells are resuspended in a carbonate-buffered sucrose solution to obtain protoplasts (3). An aliquot of the plasmid DNA to be transformed is diluted in sucrose solution, mixed with liposome DOTAP reagent and incubated to allow the formation of DNA-liposome complexes. Then 990 µl of *M. mazei* protoplasts are added to this DNA-liposome mixture and incubated at 37 °C for 4–6 h (4). A 500-µL portion of the transformation mixture is added to 5 mL of minimal medium supplemented with sucrose and further incubated for 12–16 h (5). Then, 300 µL of the culture is transferred into 5 mL of minimal

medium containing puromycin to select for puromycin-resistant clones (6). When growth and gas formation gets visible, 200 µL of the culture is transferred to minimal media containing no sucrose allowing the s-layer and cell wall to fully reform before plating (7). These cultures are finally plated on agar (8) and single colonies can be obtained after 1-2 weeks (9). Created with BioRender.com.

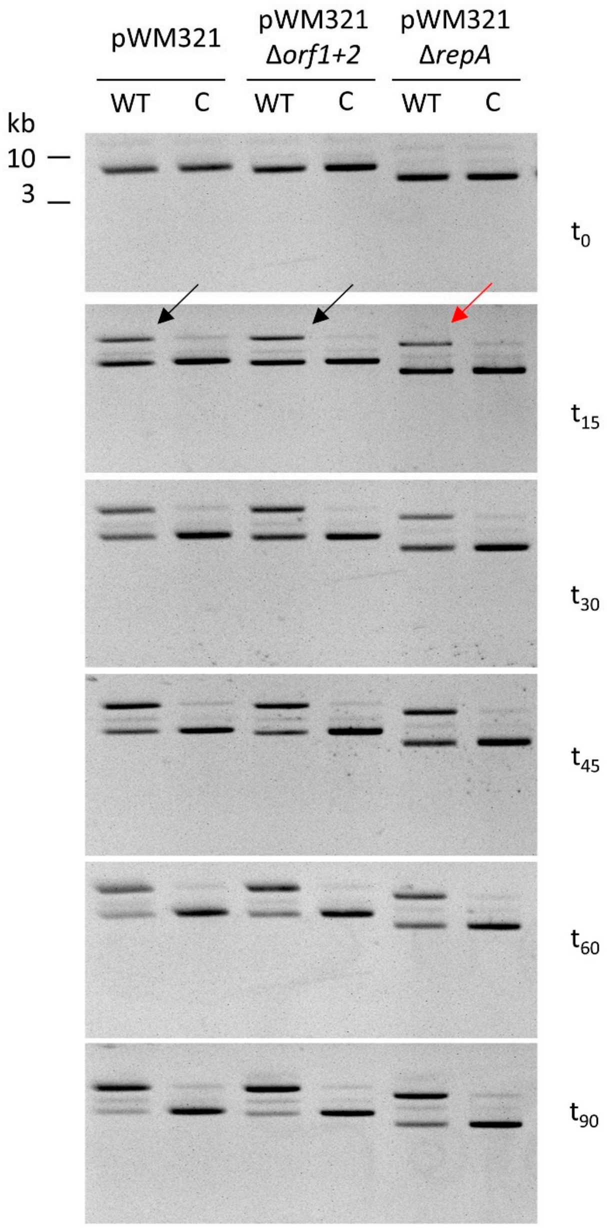

Figure S2: Nicking of different derivatives of pWM321. The nicking activity of RepA was compared for pWM321, pWM321Δ*orf1*+2 and pWM321Δ*repA*. In each case, 100 ng of plasmid and 20 ng of protein was used in 10 mM Tris (pH 6.9, 10 mM $MgCl_2$). The mixtures were incubated at 37 °C and samples were taken after 0, 15, 30, 45, 60 and 90 minutes. After 15 minutes a nick is induced for all tested plasmids (indicated with arrows), with pWM321Δ*repA* being cut less efficiently (red arrow).

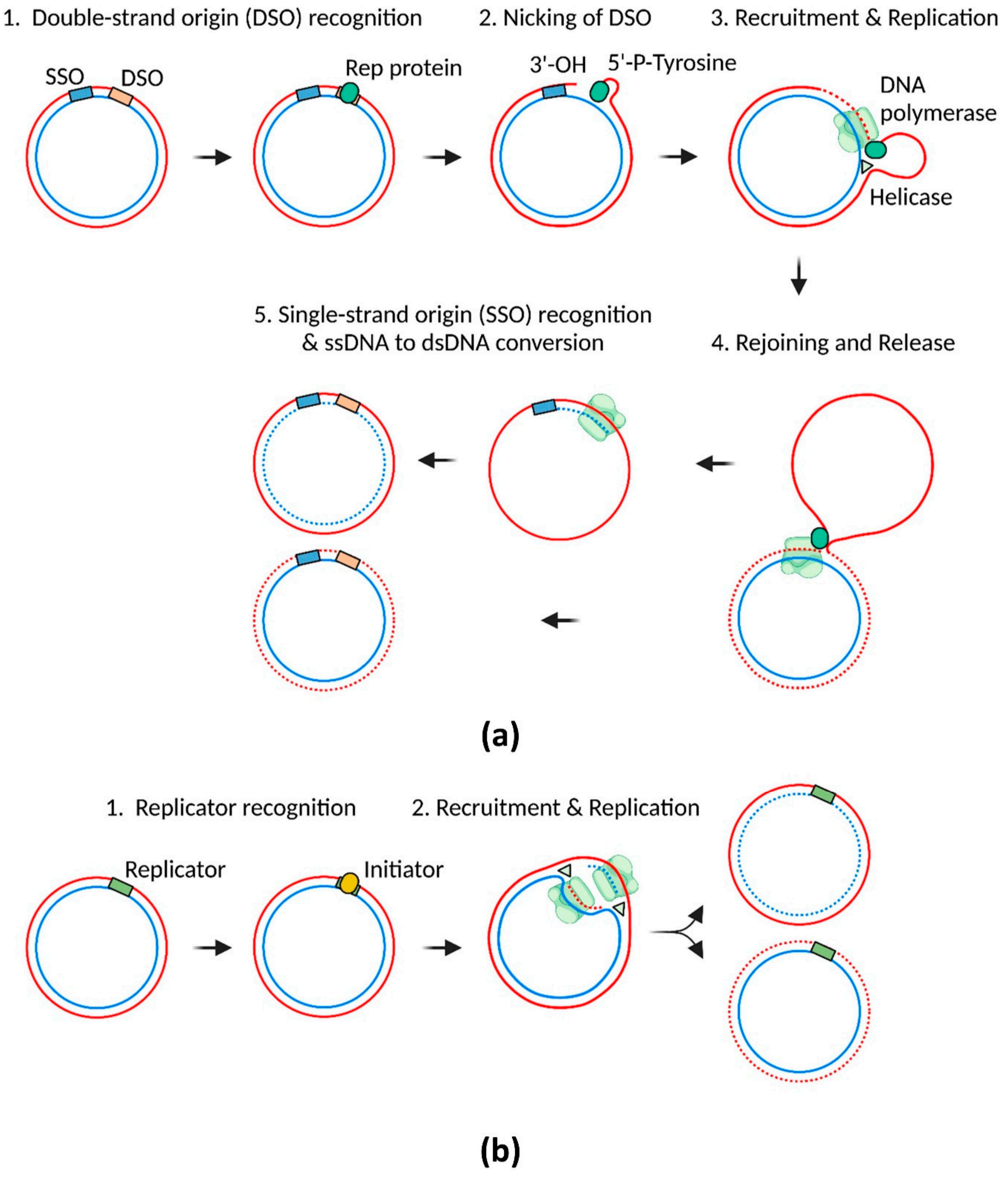

Figure S3: Plasmid replication mechanisms. (a) Rolling circle replication starts with the recognition of the double-strand origin (DSO) and the binding of the rep protein (1). The active centre of the rep protein usually carries a tyrosine that catalyses a nick of the dsDNA and binds the 5′ end resulting in a free 3′ end as a start point for elongation (2). After recruitment of the DNA polymerase, helicase and other interaction partners, the rep type protein travels along with the replication fork and, *in vivo*, being bound to the ssDNA through the 5′-phosphotyrosine bond (3). When elongation is complete, the active site tyrosine is involved in re-joining the 3′ and the 5′ end with an esterification, so that one dsDNA and one ssDNA molecule is released and a host RNA polymerase or primase is recruited (4). In the last step, starting at the single-strand origin (SSO), the ssDNA is converted to dsDNA, resulting in two

identical dsDNA plasmid copies (5). (b) Bidirectional replication also starts with recognition of the origin by an initiator protein (1). Afterwards the dsDNA melts at the replicator site creating two single strands as start points for replication. Replication can then take place either in theta mode, where DNA synthesis is continuous on one of the strands (leading strand) and discontinuous on the other (lagging strand) or in strand displacement mode, where replication proceeds continuously on both strands (2). Created with BioRender.com.
